# Large Area Few-Layer Hexagonal Boron Nitride as a Raman Enhancement Material

**DOI:** 10.3390/nano11030622

**Published:** 2021-03-02

**Authors:** Nilanjan Basu, Moram Sree Satya Bharathi, Manju Sharma, Kanchan Yadav, Avanish Singh Parmar, Venugopal Rao Soma, Jayeeta Lahiri

**Affiliations:** 1 School of Physics, University of Hyderabad, Hyderabad 500046, India; nilanjanbasu85@gmail.com; 2 Advanced Centre of Research in High Energy Materials (ACRHEM), University of Hyderabad, Hyderabad 500046, India; mssbharathi@uohyd.ac.in; 3 School of Chemistry, University of Hyderabad, Hyderabad 500046, India; manjusharma@uohyd.ac.in; 4 Department of Physics, Indian Institute of Technology (BHU), Varanasi 221005, India; kanchany10038@gmail.com (K.Y.); asparmar.phy@itbhu.ac.in (A.S.P.); 5 Department of Physics, Banaras Hindu University, Varanasi 221005, India

**Keywords:** 2D materials, hBN, CVD, SERS, FESEM, AFM

## Abstract

Increasingly, two-dimensional (2D) materials are being investigated for their potential use as surface-enhanced Raman spectroscopy (SERS) active substrates. Hexagonal Boron Nitride (hBN), a layered 2D material analogous to graphene, is mostly used as a passivation layer/dielectric substrate for nanoelectronics application. We have investigated the SERS activity of few-layer hBN film synthesized on copper foil using atmospheric pressure chemical vapor deposition. We have drop casted the probe molecules onto the hBN substrate and measured the enhancement effect due to the substrate using a 532 nm excitation laser. We observed an enhancement of ≈10^3^ for malachite green and ≈10^4^ for methylene blue and rhodamine 6G dyes, respectively. The observed enhancement factors are consistent with the theoretically calculated interaction energies of MB > R6G > MG with a single layer of hBN. We also observed that the enhancement is independent of the film thickness and surface morphology. We demonstrate that the hBN films are highly stable, and even for older hBN films prepared 7 months earlier, we were able to achieve similar enhancements when compared to freshly prepared films. Our detailed results and analyses demonstrate the versatility and durability of hBN films for SERS applications.

## 1. Introduction

Surface-enhanced Raman spectroscopy/scattering (SERS), an offshoot of Raman spectroscopy, is commonly used in chemical sensors to detect trace quantities of analyte molecules even down to single molecules [1,2]. In this technique, when analyte molecules adsorb on the SERS substrate, its Raman signal is amplified such that the signal intensity becomes comparable to that of fluorescence. The two widely accepted mechanisms behind SERS enhancement are electromagnetic enhancement (EM) and chemical enhancement (CM). The EM enhancement has contributions from (i) the local field enhancement produced by the excitation of surface plasmons and (ii) the re-radiation enhancement [3]. The EM enhancement can result in an enhancement factor as high as 10^8^–10^11^ and is independent of the nature of the analyte molecule. The chemical enhancement is strongly dependent on the chemical nature of the analyte molecule. It has contributions from (i) charge transfer resonances between the substrate and the adsorbed analyte and/or (ii) adsorption-induced non-resonant modifications of molecular polarizability of the analyte molecule [3,4]. The contribution of the chemical enhancement is much smaller than the electromagnetic enhancement, and its magnitude may reach 10^2^–10^4^. Commonly used SERS substrates are roughened noble metal substrates (Ag, Au, Cu), metal nanoparticles in suspension and metal nanoparticles immobilized on substrates [5]. In these metal-based substrates, the electromagnetic enhancement is so strong that it overshadows any chemical enhancement effect. Metallic substrates also suffer from some drawbacks such as low oxidation resistance, catalytic activity, and photobleaching effect [6,7]. To overcome these problems passivating or encapsulation layers composed of inert materials such as ultra-thin Al_2_O_3_, SiN, dithiocarbamate, and other dielectric films are used [8,9]. Semiconductors and dielectrics have also been explored as alternative SERS active substrates [4]. Recently, two-dimensional (2D) materials are being investigated for next generation of SERS substrate [10,11,12,13],. Two-dimensional (2D) materials have large specific surface area, high oxidation resistance, high chemical inertness, high thermal conductivity, high flexibility, and good biocompatibility, which are all highly desirable features required for SERS substrates [14,15]. In addition, the large-scale uniform coatings of 2D materials can be synthesized using controllable and cost-effective methods, thus ensuring a better reliability and reproducibility of Raman signals. In 2D materials, CM is the dominant mechanism of enhancement, the enhancement factors (EFs) are sensitive to the molecule type, molecule orientation, molecule concentration, laser wavelength, and substrate type [16,17].

In this work, we have investigated the SERS activity of few-layer hexagonal Boron Nitride (hBN) films. hBN, an analogue of graphene, has a similar layered structure with Boron and Nitrogen atoms arranged in a hexagonal lattice. Graphene has a covalent C-C bond, while hBN has a polar B-N bond. hBN is also an electrical insulator with a band gap of 6 eV [17]. It has an atomically smooth surface free of dangling bonds and high thermal conductivity [18]. It is a metamaterial that has found its way to an array of applications [19]. Exfoliated hBN has also been used as an effective insulating and passivating layer for SERS substrates [20,21,22]. Its exceptional chemical inertness ensures good stability against photothermal and oxidative damages, and its high thermal conductivity helps in heat dissipation. Previous literature on the surface-enhanced Raman spectroscopy (SERS) about hBN mostly deals with composite materials using exfoliated hBN flakes as a passivating layer for metal nanoparticles [6,23,24]. We report the use of large-area ultra-thin hBN films synthesized by atmospheric pressure chemical vapor deposition (APCVD) as the SERS active substrate. We used three standard dye molecules—methylene blue (MB), malachite green (MG), and Rhodamine 6G (R6G) with high Raman scattering cross-section as probe molecules. We observed enhancement of the order ≈10^3^ for MG and ≈10^4^ for 1 μM concentration of MB and R6G dyes. We also demonstrated the effect of film thickness and film morphology on the Raman enhancement of MB molecules. The enhancement observed due to these films is comparable to that of exfoliated hBN flakes. We were able to reuse the hBN substrates multiple times for SERS measurements after cleaning the substrates with ethanol without any significant loss in sensitivity. The chemical inertness of hBN films ensured that we were able to observe the same order of enhancement even for films synthesized seven (7) months apart. The SERS enhancement could be reliably reproduced for different hBN films synthesized in different batches, thereby demonstrating the versatility of hBN as SERS substrates.

## 2. Materials and Methods 

### 2.1. Materials

Dye molecules methylene blue (MB—C_16_H_18_ClN_3_S), malachite green (MG—C_23_H_25_ClN_2_), and Rhodamine 6G (R6G—C_28_H_31_N_2_O_3_Cl) were purchased from M/s Sigma Aldrich (St. Louis, MI, USA) and used as is.

### 2.2. Synthesis of hBN Films

Hexagonal boron nitride has been synthesized using APCVD on a copper substrate (99.8%, 25 μm thick, M/s Alfa Aesar, Tewksbury, MA, USA). Ammonia Borne (AB) powder was used as a precursor material (Sigma Aldrich, St. Louis, MI, USA). A mixture of Argon and Hydrogen (9:1, 99.99% purity) was used as a carrier gas during the growth time. Different amounts of AB powder were used to synthesize different thicknesses of hBN on Cu foil. The as-received Cu foil (see Figure S1) was cleaned with dilute nitric acid to remove oxides followed by rinsing in Deionised Water ater, acetone, and Isopropyl alcohol (Sisco Research Laboratories, Maharashtra, India) and then pre-annealed at 1050 °C for one hour in Ar/H_2_ (9:1) atmosphere [24]. After this, the Cu substrate was placed at the center of a horizontal tubular furnace of 5 cm diameter and 1.2 m in length. The AB powder was placed 55 cm upstream from it. The system was purged using 200 sccm of Ar/H_2_ (90/10) gas mixture for 30 min. After purging, the Cu substrate was heated to 1050 °C with a heating rate of 8 °C/min from room temperature (RT) to 600 °C under 200 sccm Ar gas, and from 600 to 1050 °C under 200 sccm of Ar/H_2_ (9:1). Then, the gas flow rate was kept constant at 50 sccm Ar/H_2_ (9:1) until the reaction was over. The substrate was kept at 1050 °C for 30 min, and then, it was allowed to cool down naturally to RT. During the cooling phase, the gas flow rate was increased to 200 sccm Ar/H_2_ (9:1). The temperature profile used for synthesis is shown in Supplementary Figure S2. The precursor heating was initiated when the substrate temperature reached 1050 °C. For varying the thickness of hBN films, the starting amount of precursor powder was varied while keeping all the other growth-related parameters constant. For 7, 6, and 1.5 nm thick hBN film, 10.8, 9, and 4.9 mg AB powder was used, respectively. Thicker h-BN flakes were exfoliated from hBN powder (Momentive AC6004, La Rochette, France). 

### 2.3. SERS Characterization 

The Raman spectra was acquired using a Horiba Labram Raman spectrophotometer (Tokyo, Japan) with 532 nm laser, 100× objective (532 nm; estimated laser spot size of ≈720 nm) and typically 100 mW input power for all Raman measurements. For each Raman spectra, data were accumulated for 2 cycles, and the integration time was set at 5 s. All these parameters were kept constant unless otherwise mentioned. The 4 μL dye solution (in ethanol) was drop casted on hBN substrate and allowed to dry before recording Raman spectra. For enhancement factor calculations, the Raman spectra was measured on at least 10 different positions on the same sample. During each and every Raman measurement for a particular set of film thickness and dye, every aspect of the measurement such as laser power, wavelength, objective lens, grating, spectrometer, etc. all were kept the same. Raman peak position and full width half maxima (FWHM) was calculated by fitting the Raman peaks with a Lorentzian function.

### 2.4. Characterization Methods

The synthesized hBN films supported on Cu substrates were characterized by using Field Emission Scanning Electron Microscopy (FESEM), Atomic Force Microscopy (AFM), and X-ray Photoelectron spectroscopy (XPS). FESEM images were acquired using a Carl Zeiss Ultra 55 model operated at 5 kV (Carl Zeiss, Berlin, Germany). An optical microscope (Olympus, Japan) with a DP73 camera was used to record optical micrographs. AFM studies were done with a SEIKO Instrument (Tokyo, Japan). X-ray photoelectron spectroscopy was recorded using an Axis Ultra system equipped with a monochromatic Al Kα X-ray source (1486.6 eV) with 0.1 eV energy resolution (Shimadzu, Kyoto, Japan). The XPS spectra were analyzed using CASA XPS software. The peaks were fitted with a Gaussian Lorentzian function, and the peak area was calculated. The stoichiometry of the film was calculated by taking the ratio of the N1s and B1s area under the peak corrected by Relative Sensitivity Factors (RSF). The hBN film was transferred onto a SiO_2_/Si substrate by using the wet etching method with 1 M FeCl_3_ solution [25]. Transmission Electron microscopy (TEM) analysis was done using a FEI Tecnai G2 20 TWIN transmission electron microscope (FEI, New York, USA), which has been used to obtain the Small Area Electron Diffraction (SAED) pattern. The aperture was 20 µm, and accelerating voltage was 200 kV. The film from the copper substrate was transferred over a copper grid with carbon nanomesh by the wet transfer method, which has been applied in previous transfers. 

### 2.5. Simulation Details

The calculations were performed using a Quickstep module of CP2K software using the Perdew-Burke-Ernzerhof (PBE) exchange–correlation functional [26,27,28]. The double-zeta plus polarization quality DZVP basis set and Goedecker-Teter-Hutter pseudopotential (DZVP-GTH-PADE) were chosen for the hBN layer, and molecularly optimized basis sets DZVP-MOLOPT-SR-GTH were used for the dye molecules [29]. The norm-conserving Goedecker–Teter–Hutter pseudopotentials were employed for scalar–relativistic core corrections [30]. The cutoff for kinetic energy was set to 280 Ry, and three-dimensional periodic boundary conditions were applied. The Grimme’s DFT-D3 van der Waals corrections were chosen to describe the dispersion interactions [31]. Born–Oppenheimer molecular dynamics simulations were performed at 300 K with a timestep of 1.0 fs using a Noose–Hover thermostat for the NVT ensemble [32]. The systems were equilibrated for 2 ps, and final configurations were used to calculate the hBN–dye interaction energy as $E_{r}=E_{complex}-E_{dye}-E_{hBN}$. The dimension of the chosen single hBN layer was 3.2 nm × 2.0 nm × 3.0 nm. The effect of the system size was tested by simulating all the systems with a 3.2 nm × 2.0 nm × 4.0 nm simulation box, and the results show good agreement for hBN–dye interaction energies for both the system sizes, as reported in Table S1. The terminal boron atoms were saturated with hydrogen atoms (number of atoms in the layer; $N_{B}=N_{N}=71,N_{H}=16$). We generated three systems where each system consisted of 1 molecule of dye (MB, MG, and R6G) and a single hBN layer.

## 3. Results and Discussion

The ultra-thin hBN films were characterized using several techniques and were investigated for SERS activity.

### 3.1. hBN Film Characterization

**Figure 1:**
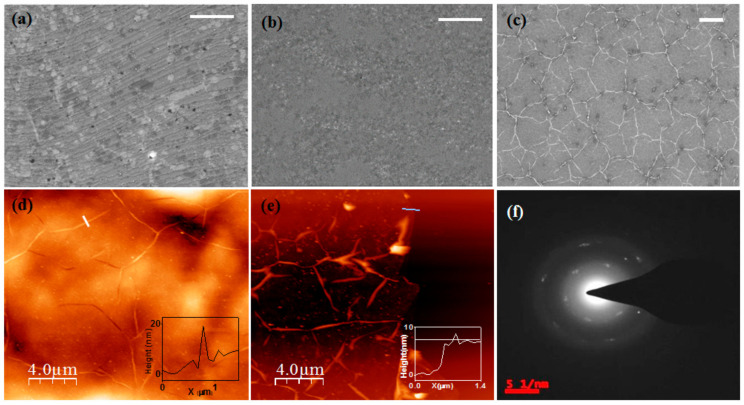
Field Emission Scanning Electron Microscopy (FESEM) and Atomic Force Microscopy (AFM) studies of the as-synthesized film. (**a**–**c**) FESEM images of the hexagonal Boron Nitride (hBN) film over a Cu substrate of 1.5, 6, and 7 nm film. Scale bar −2 µm (**a**,**b**), 5 µm for (**c**). (**d**) AFM micrograph of the 7 nm film over copper substrate, Inset—Height profile of the wrinkle. (**e**) AFM image of the transferred 7 nm film (the mean height of the film is 7 nm), Inset—Height profile. (**f**) Selected area electron diffraction (SAED) pattern of the film (6 nm) at 200 kV, depicting polycrystalline nature.

Figure 1a–c illustrate the FESEM micrographs of three different hBN films deposited using 4.9, 9, and 10.8 mg AB powders, respectively. The hBN films in Figure 1a,b are very flat, while the film morphology in Figure 1c is very different, with the surface covered a network of bright lines all over the surface. These bright lines correspond to the out-of-plane deformation of hBN film where it gets locally delaminated from the substrate to form a wrinkle kind of structure. These wrinkles are a ubiquitous feature found in films of 2D materials (such as graphene, MoS_2_) synthesized by Chemical Vapor Deposition (CVD) on metal substrates. This is a consequence of the mismatch of the thermal expansion coefficient (TEC) between the substrate and the film [33]. Unlike few layer graphene, few layer hBN with partial ionic bonding has a higher bending rigidity and is less likely to form out-of-plane deformations. However, from FESEM, we observe the presence of a large number of wrinkles and folds in the films with the density of wrinkles/folds increasing with film thickness. Our observation is similar to those reported for wrinkled hBN film on sapphire synthesized by Metalorganic Vapor Phase Epitaxy (MOVPE) [34]. The authors here found a higher mean residual compressive strain in thicker films but did not elucidate on the origin of such a higher strain in thicker film. Figure 1d shows the AFM image of the hBN film supported on Cu foil as shown in panel Figure 1c. From the AFM images, we observe that most of these wrinkles have an average height and width as 17 nm ± 1 nm and 226 ± 44 nm, respectively. The film thickness was determined from the edge thickness measurement using AFM. For edge thickness measurement, the hBN film was transferred to the SiO_2_/Si substrate using protocol as mentioned in the experimental methods section. The hBN film (shown in Figure 1c) was transferred to SiO_2_/Si, and the AFM image of the edge is shown Figure 1e. From this AFM image, we get the thickness of the film as 7.1 ± 0.73 nm. For the hBN films shown in Figure 1a,b, we get thickness of 1.5 ± 0.11 nm, 6.1 ± 0.25 nm respectively (see Figure S3). The average film thickness and standard deviation were calculated by measuring at least at eight different locations along the edge. Figure 1f shows the Selected Area Electron Diffraction (SAED) pattern of a 6 nm thick hBN film. We observed several sets of diffraction spots with hexagonal symmetry, indicating we have a polycrystalline hBN film.

**Figure 2:**
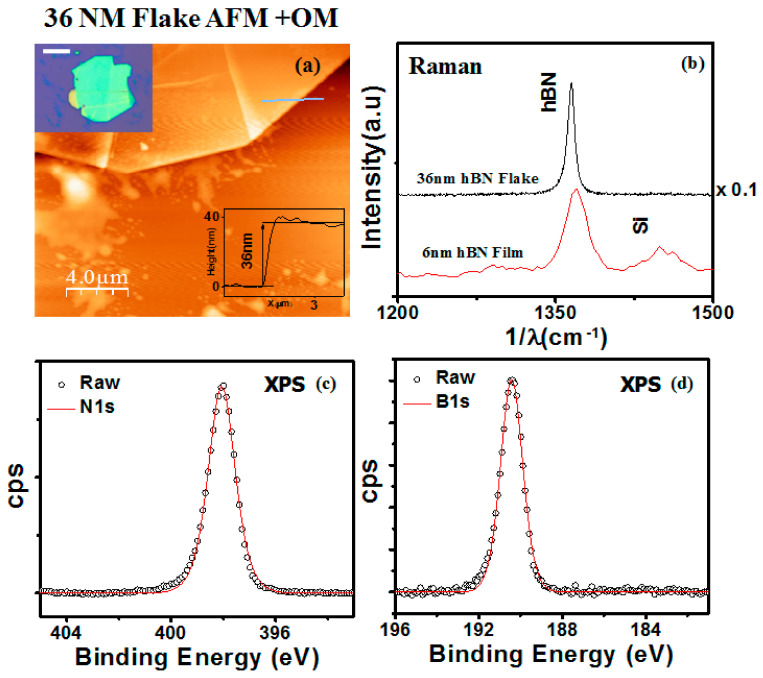
(**a**) AFM image of the flake. Inset—Height profile and Optical Micrograph (OM), Inset scale 10 μm. (**b**) Raman of the transferred 6 nm thick hBN film and exfoliated hBN flake (λ = 532 nm) (**c**,**d**) B1s and N1s high resolution XPS spectrum.

For comparison with the APCVD films, hBN flakes were also mechanically exfoliated from hBN powder. Figure 2a shows the optical and AFM image of one such exfoliated hBN flake. The thickness of this hBN flake was determined to be 36 nm. Figure 2b shows the Raman spectrum of the 6 nm hBN film transferred to a SiO_2_/Si substrate and a 36 nm thick hBN exfoliated flake. For the exfoliated hBN flake, we are able to observe a sharp Raman peak at 1365 cm^−1^ with FWHM maxima of 8 cm^−1^. This corresponds to the in-plane vibration of the boron and nitrogen atoms, the E_2g_ vibrational mode of hBN [23]. The Raman spectrum of the 6 nm of hBN film shows two peaks present at 1370 cm^−1^ corresponding to an E_2g_ peak and the other at 1450 cm^−1^, which corresponds to the third over tone of the Si [35]. The full width half maxima (FWHM) of the E_2g_ peak is 24 cm^−1^, which is the value reported for most hBN films synthesized by APCVD [36]. The FWHM of the E_2g_ peak of 1.5 nm and 7 nm thickness films were 19 cm^−1^ and 30 cm^−1^, respectively, indicating that all hBN films of different thicknesses have similar crystalline quality. To characterize the chemical composition of the hBN film, we conducted XPS measurements. The survey spectrum of the 6 nm film is shown in Figure S4. Figure 2c,d illustrate the high-resolution N1s and B1s XPS spectra of 6 nm thick film. The N1s peak 398.1 eV and B1s peak at 190.4 eV correspond to a few-layer hexagonal boron nitride film, and these values are similar to those reported in the literature [37]. The stoichiometry of the BN film was calculated as B:N = 0.94:1, which shows that the hBN film is nearly stoichiometric. We performed UV-Vis spectroscopy to find the band gap of the prepared film. Figure S5 shows the UV-Vis spectra of the 6 nm film; there is a deep UV peak at 201 nm, and the inset is the corresponding Tauc’s plot. The band gap calculated from the Tauc plot is 5.7 eV. This is fairly consistent with the prevailing literature [38,39]. 

### 3.2. SERS Measurements

**Figure 3:**
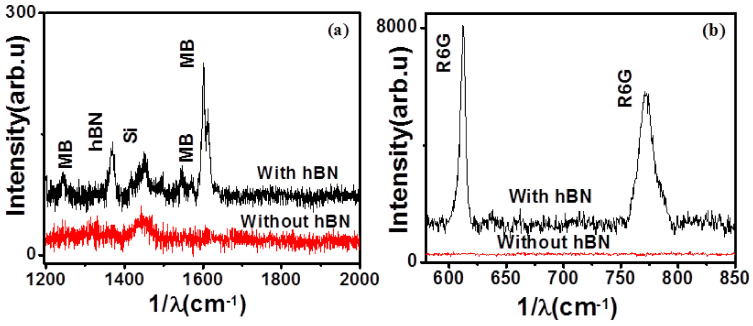
Enhancement of the Raman signal of (**a**) 1 μM methylene blue (MB) and (**b**) 50 μM Rhodamine 6G (R6G) over 6 nm hBN. The Raman spectra with hBN and without hBN are shown.

To probe Raman enhancement effects by the hBN substrate, we used three standard model dyes—methylene blue, malachite green and rhodamine 6G. The dye solutions (in ethanol) were drop casted on hBN substrate and allowed to dry; then, the Raman spectra were recorded. To compare the enhancement effect, Raman spectra of the dyes were recorded on an hBN film-coated SiO_2_/Si substrate and blank SiO_2_/Si (reference substrate). Figure 3a shows a representative Raman spectrum of 1 μM MB over 6 nm thick hBN films where we can clearly observe the enhancement effect due to the presence of hBN films. For 1 μM solution of MB, no signal was detected for MB on the SiO_2_/Si substrate, but a large signal at 1601 cm^−1^ was detected on the hBN/SiO_2_/Si substrate. Figure 3b shows the SERS spectra of 50 μM R6G with and without hBN over the SiO_2_/Si substrate. To quantify the enhancement effect of the hBN substrate, the analytical enhancement factor (AEF) was calculated as

$$AEF=\frac{I_{SERS}\;X\;C_{Raman}}{I_{Raman}\;X\;C_{SERS}}$$

where

*I_SERS_* is the Raman intensity of the probe molecule on the hBN substrate
*C_SERS_* is the corresponding concentration of the probe molecule in ethanol
*I_Raman_* is the Raman intensity of the probe molecule on reference substrate
*C_Raman_* is the corresponding concentration of the probe molecule in ethanol.

**Figure 4:**
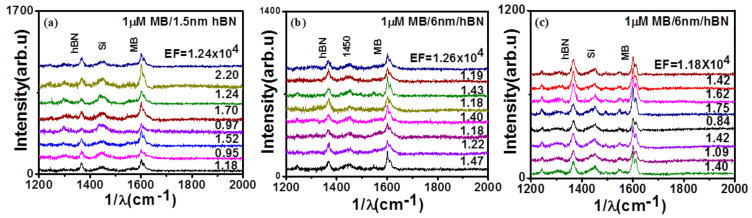
(**a**,**b**) Raman enhancement of 1 μM MB by 1.5 and 6 nm film at different positions of the film. (**c**) Reproducibility studies—Raman enhancement of 1 μM MB by a different 6 nm film at different positions.

MB has 14 distinguishable Raman peaks in the range of 250–1750 cm^−1^ with the most intense peak at 1624 cm^−1^. The band at 1618 cm^−1^ (C-C stretching) was taken as the reference for the calculation of AEFs. Figure 4 shows Raman spectra of MB on different positions for the 6 nm and 1.5 nm thick hBN, and the spectra are offset for clarity. The AEFs for 1.5 and 6 nm hBN film were calculated for over eight positions, and they varied (0.9−2.2) × 10^4^ and (1.18−1.47) × 10^4^, respectively (see data presented in Figure 4a,b). The standard deviation of AEF for 1.5 and 6 nm film was 0.4 and 0.11, and mean AEFs were 1.37 × 10^4^ and 1.29 × 10^4^, respectively. To ensure the reproducibility of our results, the Raman measurements were repeated with samples from different synthesis batches, and all of the measurements showed similar results (Figure 4c), where the mean AEF was 1.34 × 10^4^ with a standard deviation of 0.27. These measurements were also repeated for hBN films of thickness 7 nm. Figure S6 shows the enhancement at different positions for the 7 nm film. Clearly, the enhancement results are consistent with different batches of hBN for different hBN film thicknesses. The signal-to-noise (S/N) ratio can be improved by increasing the integration time further. This is an initial proof-of-concept study, and we believe there is huge scope for improving the enhancement factors.

We investigated two more organic dyes, malachite green (MG) and R6G, as a SERS probe with the 6 nm film. Malachite green has a sharp Raman band near 1617 cm^−1^, which has been used as a reference for enhancement studies [40] (see Figure S7a). Figure S7b depicts a representative Raman spectrum of 50 μM R6G over 6 nm thick hBN films where enhancement is observed for in the presence of hBN film. We estimated the analytical enhancement factor at different spots on the sample to get an average AEF 1.5 × 10^4^ for R6G and 6.44 × 10^3^ for MG (Figure S7a). This is an order less than the AEF obtained for methylene blue. The difference in the order of enhancement can be explained by the strength of interaction of dyes with hBN substrate. It is believed that the polar nature of hBN induces an interfacial dipole in the adsorbed organic molecules, which increases the transition probability that gives rise to the enhancement in Raman enhancement effect [7,14]. In this case, the interaction between hBN and methylene blue having a π-conjugated aromatic structure is primarily determined by the π–π interaction. For MG and R6G dyes, the interaction with hBN is relatively weaker, resulting in lower enhancements. First principles-based Molecular Dynamics (MD) calculations were performed to gain better understanding of the interaction of these dyes with hBN, which is discussed in Section 3.3. 

**Figure 5:**
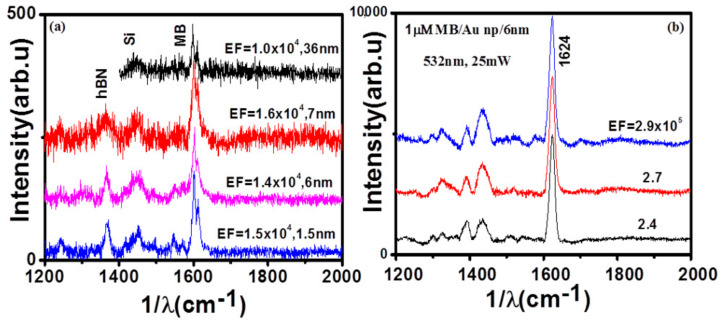
(**a**) Comparison of Raman enhancement of 1 μM MB using CVD hBN films of thickness 1.5, 6, and 7 nm film and exfoliated hBN flakes of 36 nm thickness. (**b**) Raman enhancement of 1 micromolar MB with Au nanoparticles and hBN.

**Figure 6:**
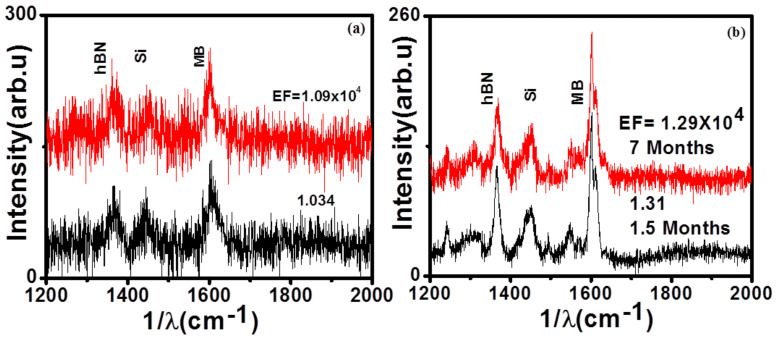
(**a**) Raman enhancement after regenerating hBN films: (**a**) 1 uM MB on 7 nm thick hBN film and (**b**) 1 μM MB by 6 nm film at different time durations after their growth.

Figure 5a shows the Raman spectra of 1 μM MB on hBN films of different thicknesses. For the hBN films synthesized using CVD, the calculated average EFs for 1.5, 6, and 7, films were 1.37 × 10^4^, 1.29 × 10^4^, and 1.5 × 10^4^, respectively. The MB peak splits into two peaks centered at 1601 cm^−1^ and 1612 cm^−1^ and down shifts. This down shift and splitting of the 1618 cm^−1^ peak are evident in both the CVD-prepared film and in the hBN flake. This shift and splitting of the 1618 cm^−1^ peak has been reported in earlier SERS studies of MB [41]. The calculated AEFs were similar for 1.5, 6, and 7 nm thick hBN films, indicating that the film thickness had no impact on the enhancement factors for the hBN film, which is similar to previously reported results. Ling et al. had observed no change in enhancement factor for mechanical hBN flakes of different thickness, since there was no absorption of light in the visible region [7]. We have also compared the enhancement effect of our hBN films synthesized by APCVD with mechanically exfoliated hBN flakes. The 36 nm thick hBN nanosheet was exfoliated from hBN powder and used for SERS measurement. For 1 μM MB dye, we also observed similar degree of enhancement to that of 1.5, 6, and 7 nm films synthesized by APCVD film. The surface morphology (roughness) usually plays an important role in creation of local hot spots, which leads to even higher enhancement of the Raman signal of the adsorbed analyte [42,43]. Interestingly, for the case of a highly wrinkled surface of 7 nm thick hBN film (with highest surface roughness), we observed similar enhancement of ≈10^4^ for MB molecules compared to the flat hBN films. Even though the enhancement factors are not comparable to those of plasmonic substrates, they are found to be superior than other 2D materials such as MoS_2_ (see Table S1). The enhancement can be further improved by using hBN in conjunction with Au nanoparticles. We demonstrated this by using a hybrid substrate with Au nanoparticles deposited on hBN films and then measured the SERS signal of MB. Figure 6 shows the Raman spectra of MB on a hybrid Au/hBN substrate for 532 nm laser excitation. We estimated mean AEF to be 2.6 × 10^5^. For 633 nm excitation, the mean enhancement factor increased by one order to 1.3 × 10^6^ (see Figure S8). These values are found to be similar to those reported earlier [6].

In addition to enhancement factors, stability and reusability are some of the important parameters to keep in mind while proposing a new SERS substrate for real-world applications. Figure 6a shows the Raman spectra of MB on a 7 nm thick hBN film after cleaning with alcohol. It shows the same enhancement, which indicates that the substrate can be regenerated without any loss of sensitivity. A regenerative study was also carried out with the 6 nm thick hBN film for MG dye. Here, also after washing the dye from the 6 nm hBN film, we were able to reproduce enhancement results, and we obtained enhancement factors of the order ≈10^4^ (See Figure S9). We have also performed Raman enhancement measurements at different time spans after the growth of 6 nm thick hBN films (after 1.5 months and 7 months), as shown in Figure 6b. We observed a similar order of enhancement (≈10^4^) compared to freshly prepared hBN films. It shows that these hBN films are highly stable in ambient atmosphere. Compared to plasmonic substrates that are prone to oxidation (especially silver-based), our data suggest that hBN films tend to have a long shelf life. Thus, hBN films being chemically inert and very resistant to oxidation can be regenerated and reused multiple times over a large time duration. Thus, our results show APCVD-synthesized large area hBN films to be highly reliable and durable SERS substrates that can be used in sensing applications. The utility of hBN can further be improved by functionalization and optimization of the growth conditions.

### 3.3. First Principles-Based MD Calculations

**Figure 7:**
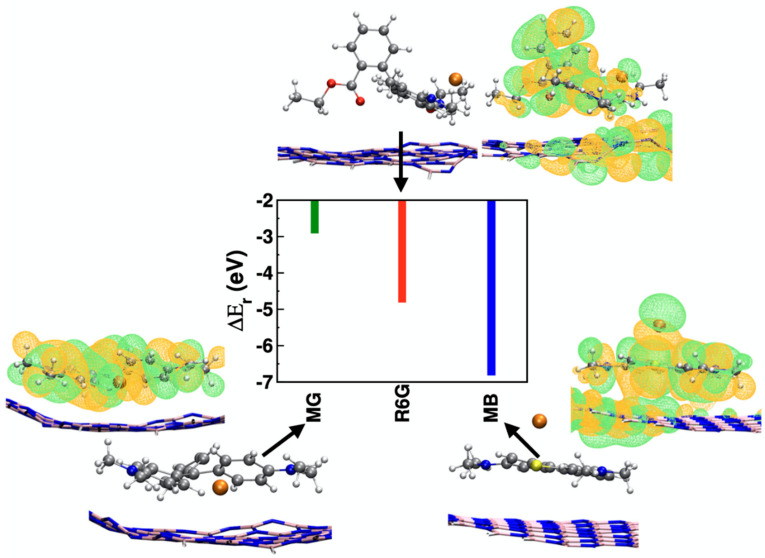
Relative interaction energy, E_d_ between a single layer of hBN and dye molecules that are MB, R6G and MG: methylene blue, rhodamine 6G, and malachite green, respectively. The gray, white blue, pink, crimson, and red colors represent C, H, N, B, Cl, and O atoms, respectively. The HOMO electron density surfaces of the complexes are plotted for an isodensity value of 0.007 a.u. (full view images are available in Figures S10 and S11).

The enhancement factor is strongly dependent on the substrate–molecule interactions. The interaction energy between single-layer hBN and different dye molecules were calculated using first principle molecular dynamics simulation techniques. The interaction of an hBN layer is energetically more favorable with MB (−6.8 eV) rather than R6G (−4.8 eV) and MG (−2.9 eV), as shown in Figure 7. The methylene blue molecule shows parallel orientation to the hBN layer as compared to the inclined orientation of MG and R6G molecules to the hBN layer, respectively. This occurs due to the rotational flexibility of benzene rings and steric hindrance of side groups, dimethyl amine (MG and R6G), and the ester group (R6G). Furthermore, the interaction between R6G and hBN is further enhanced over MG and hBN due to the presence of ester functionality where carboxylate oxygens show an interaction with the hBN layer. The effective overlap between the orbitals is higher in MB than in R6G and MG due to its parallel orientation that adopts an inclined orientation, as shown in the HOMO electron density profiles.

## 4. Conclusions

We have successfully demonstrated the Raman enhancement capability of few-layer APCVD-grown hexagonal boron nitride in the cases of methylene blue, R6G, and malachite green dissolved in ethanol. All sets of 1.5, 6, and 7 nm thick hBN films exhibited similar enhancement factors. The enhancement factors are in agreement with the theoretically calculated interaction energies of dyes with a single hBN layer (MB-hBN > R6G-hBN > MG-hBN). The MB molecule adopts parallel conformation as compared to R6G and MG molecules that show inclined conformations on the surface of hBN. The Raman enhancement from hBN is found to be independent of the thickness of the film. In our case, hBN films synthesized by APCVD were of high quality, and their enhancement effect is comparable to that of exfoliated hBN flakes. Our result provides valuable insights for understanding the Raman enhancement by hBN films and would thus be useful for developing hBN-based SERS substrates. However, additional optimization studies (in terms of film morphology, growth conditions, thickness, film smoothness, etc.) are required before we can propose hBN for practical applications. 

## Data Availability

The data presented in this study are available on request from the corresponding author.

## Supplementary Materials

The following are available online at https://www.mdpi.com/2079-4991/11/3/622/s1, Figure S1: FESEM image of the as-received Cu substrate. Figure S2: Heating profile for growth of hBN film. Figure S3: (a and b) Thickness measurement of 1.5 nm and 6 nm. Figure S4: XPS Survey spectrum of 6 nm hBN film. Figure S5: UV-Visible spectrum of the transferred film, Inset- Tauc’s plot showing band gap of 5.7 eV. Figure S6: Repeatability study with the 7 nm film—Raman enhancement of 1 μM MB by a different 7 nm film at different positions. *50 mW laser power, 532 nm laser, 100× objective, integration time—5 s, Accumulation time—2. Figure S7: (a) Raman spectrum 1 µM MG/6 nm hBN/SiO2/Si (b) Raman spectrum 50 µM R6G/6 nm hBN/SiO2/Si (c) Raman spectrum of 1 µM MB /7 nm hBN film after cleaning in ethanol for 45 min at 10 positions. Apart from the hBN and Si peak no other peak can be seen. * For all the Raman measurements in this slide—532 nm excitation, 100× objective, integration time—5 s, Accumulation—2, input laser powers of 100 mW for (a) & (c) and 25 mW were used for data presented in for the data presented in (b). Figure S8: Raman enhancement of 1 micro molar MB with Au nano particles and hBN 633 nm excitation with 10 mW. Figure S9: (a) Raman spectrum of the 1 μM MB/6 nm hBN after washing in ethanol for 45 min. (b) Re-drop casting 1 μM MB over the film in (a). Clearly regeneration has been achieved. Figure S10: Full view of the optimized geometries of complexes of a single layer of hBN and dye (a) methylene blue (MB), (b) Rhodamine 6 G (R6G) and malachite green (MG). Figure S11: Full view of HOMO electron density surfaces for isodensity value of 0.007 a.u. of the optimized geometries of complexes of a single layer of hBN and dye (a) methylene blue (MB), (b) Rhodamine 6G (R6G) and malachite green (MG). Table S1: Interaction energies of MB, R6G and MG with hBN. Table S2: Comparison of Raman enhancement of hBN films with other 2D materials.
