# Supplementary material for: Large Area Few-Layer Hexagonal Boron Nitride as a Raman Enhancement Material
Source: Nanomaterials (Basel). 2021 Mar 2;11(3):622. doi: 10.3390/nano11030622 (PMC7998565; doi:10.3390/nano11030622)
Supplement: Supplementary file 1 [file nanomaterials-11-00622-s001.pdf]

nanomaterials

Supporting Information for

Large Area Few-layer Hexagonal Boron Nitride as a Raman Enhancement Material

Nilanjan Basu [1], Moram Sree Satya Bharathi [2], Manju Sharma [3], Kanchan Yadav [4], Avanish Singh Parmar [4], Venugopal Rao Soma [2,] and Jayeeta Lahiri [1,5,*]

[1] School of Physics, University of Hyderabad, Hyderabad 500046, India; nilanjanbasu85@gmail.com (N.B.); jlsp@uohyd.ac.in (J.L.);
[2] Advanced Centre of Research in High Energy Materials (ACRHEM), University of Hyderabad, Hyderabad 500046, India; mssbharathi@uohyd.ac.in (M.S.S.B.); soma_venu@uohyd.ac.in (V.R.S.)
[3] School of Chemistry, University of Hyderabad, Hyderabad 500046, India; manjusharma@uohyd.ac.in (M.S.);
[4] Department of Physics, Indian Institute of Technology (BHU) Varanasi 221005, India; kanchany10038@gmail.com (K.Y.); asparmar.phy@itbhu.ac.in (A.S.P.)
[5] Department of Physics, Banaras Hindu University, Varanasi 221005, India; jl.physics@bhu.ac.in (J.L.)
* Correspondence: jlsp@uohyd.ac.in or jl.physics@bhu.ac.in

1. **FESEM image of as-received Cu foil used as substrate**

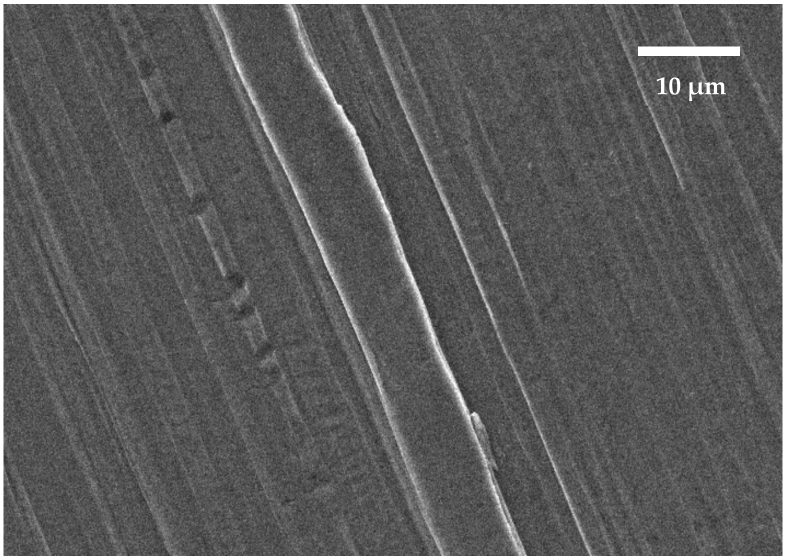

Figure S1: FESEM image of the as-received Cu substrate.

2. **Heating Profile-**The heating profile as similar for every batch of hBN synthesis. Single zone furnace from AntsLab is used for the growth with ±1 °C variation. The ammonia borane powder was sublimated by using a heating tape regulated by a variac at 130°C ±2°C for all experiments. Variac was kept at 120V.

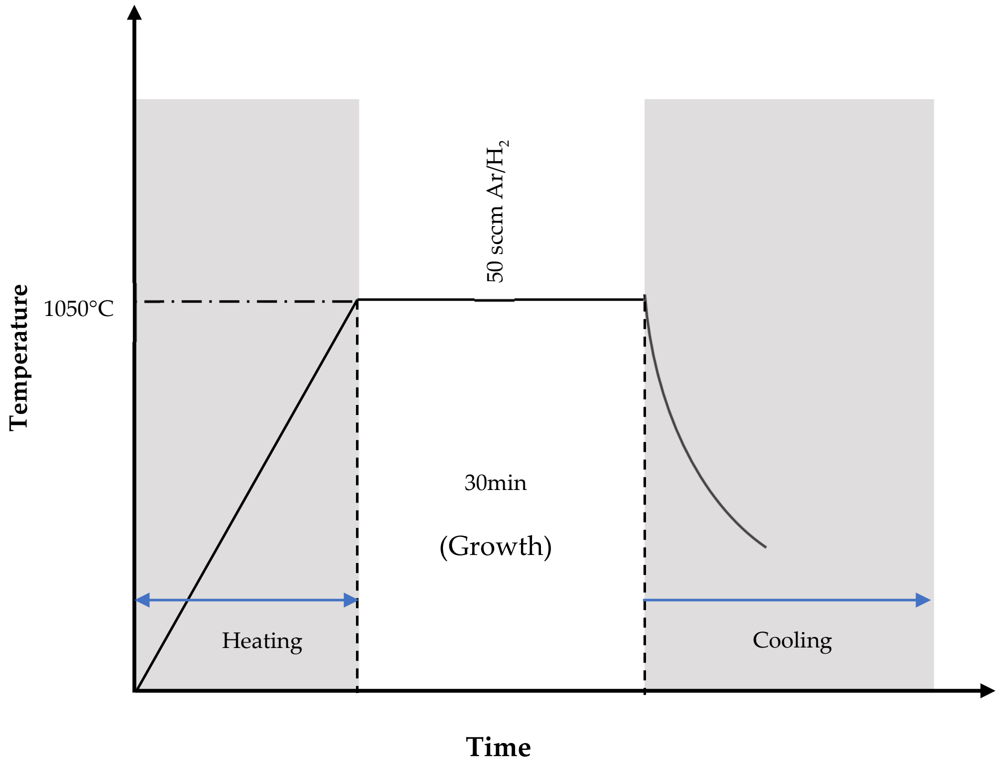

Figure S2: Heating profile for growth of hBN film.

3. Simulation Details

The effect of the system was also tested by simulating all the systems with larger box dimensions (Set 2) of 3.2 nm x 2.0 nm x 4.0 nm. The total energy ($E_{complex}$) of the complexes for all the systems is consistent with Set 1 (3.2 nm x 2.0 nm x 3.0 nm) and has an error of +/- 0.001 Hartree, as shown in the Table S1.

Table S1: Interaction energies of MB, R6G and MG with hBN

**System**	**$E_{complex}$ (Hartree) Set 1**	**$E_{complex}$ (Hartree) Set 2**
**hBN + MB**	-1077.257814077	-1077.257582858
**hBN + R6G**	-1181.169304485	-1181.171273989
**hBN + MG**	-1100.931727326	-1100.932009555

4. Thickness measurement- Thickness of 1.5nm and 6 nm has been measured at the clean edges where there in minimal leftover PMMA residue.

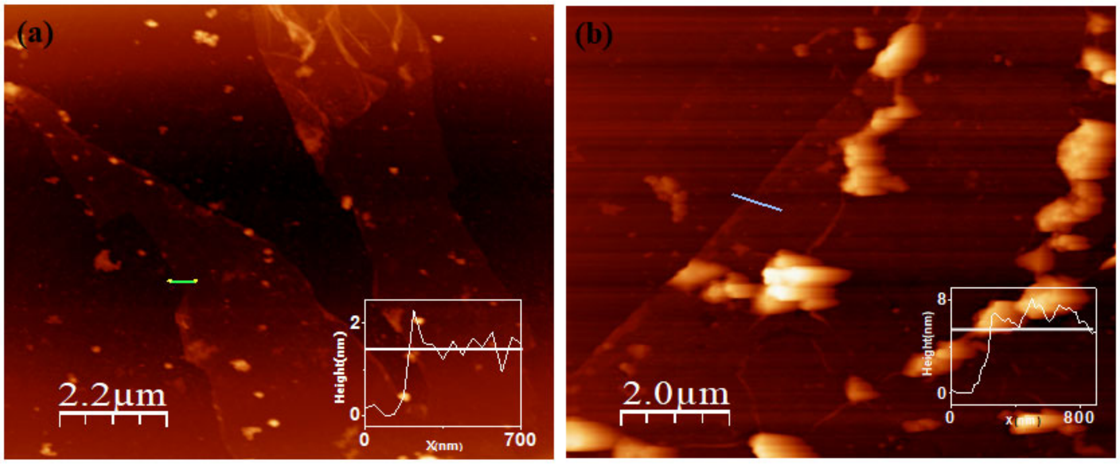

Figure S3: (a and b) Thickness measurement of 1.5nm and 6nm

5. Survey spectrum- Survey spectrum has been recorded with 1eV resolution. For both of the scans similar parameters are used scan time and number of iteration. The substrate signal which the copper signal is weak for the 6nm film. This is clearly evident for Cu 2s peak.

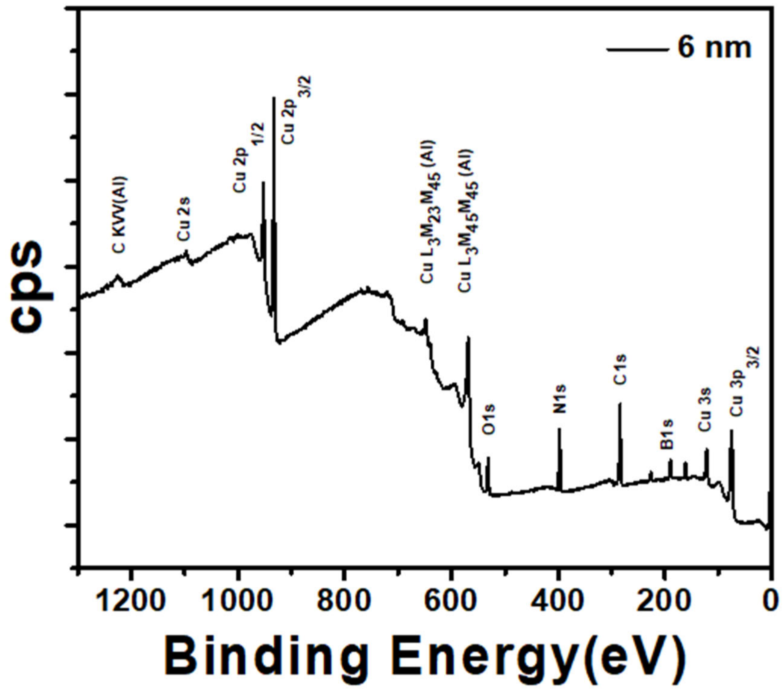

Figure S4: XPS Survey spectrum of 6 nm hBN film.

6. UV-Visible spectrum – UV-Vis spectrum is recorded by transferring the 6nm film over a 5x5mm quartz plate. The transfer process is similar to that of the film applied for SERS measurement.

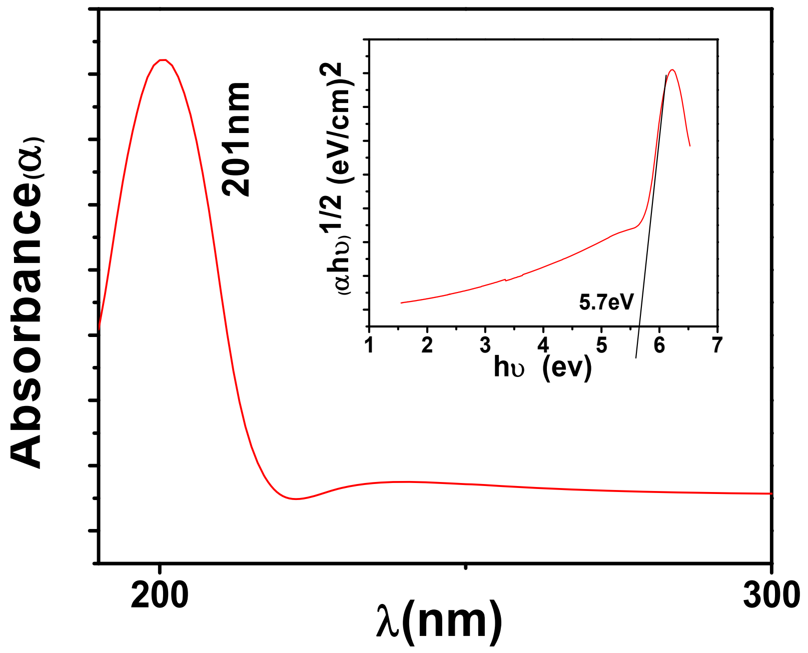

Figure S5: UV-Visible spectrum of the transferred film, Inset- Tauc's plot showing band gap of 5.7 eV.

7**. Repeatability study-** Apart from using the 6 nm and 1.5 nm film for repeatability study, we also used the 7 nm film. 50 mW laser power was used to record Raman signal from different positions of the 7 nm film.

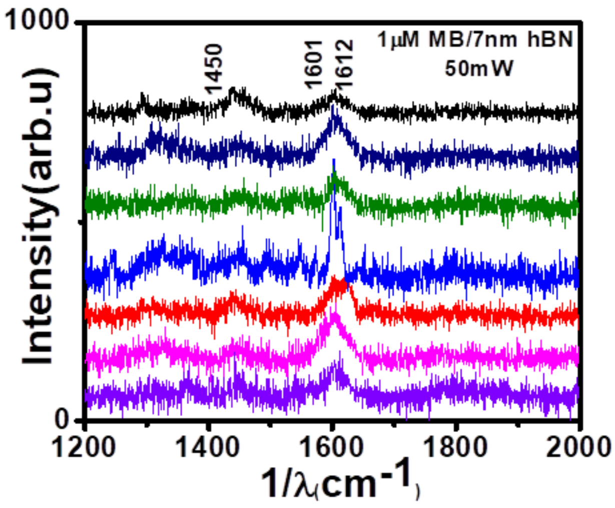

Figure S6: Repeatability study with the 7 nm film - Raman enhancement of 1 μM MB by a different 7 nm film at different positions. *50 mW laser power, 532 nm laser, 100X objective, integration time -5s, Accumulation time- 2,.

8. Malachite green (MG), Rhodamine 6G (R6G) - Raman enhancement studies were performed by using the 6nm film over MG and R6G. At all three positions of MG AEF of the order of ~10^3 has been achieved and similarly at all three positions of R6G AEF of the order of ~10^4 has been achieved. Figure S5c shows the Raman spectrum at different position of the 1 μM MB /7 nm film after washing it in ethanol for 45 min. No ultra-sonication has been done to protect the hBN film. It was simply kept in ethanol beaker for 45 min. Clearly there is no sign of MB after washing.

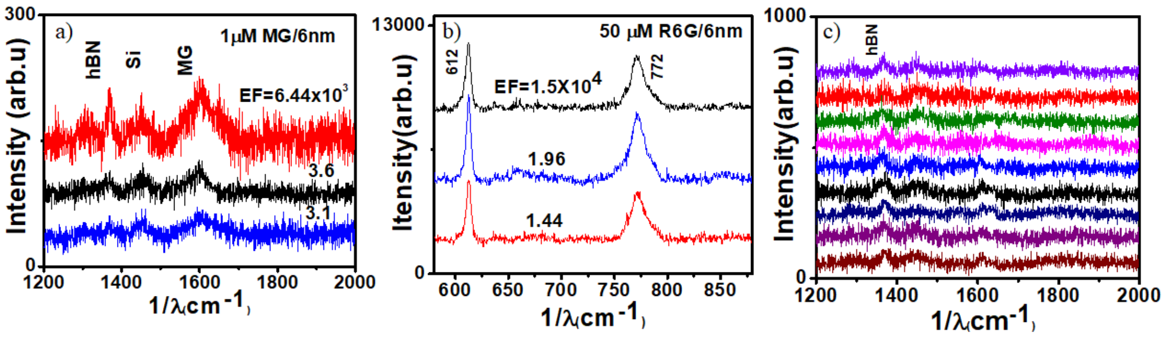

Figure S7: a) Raman spectrum 1 μM MG/6 nm hBN/SiO_2/Si b) Raman spectrum 50 μM R6G/6 nm hBN/SiO_2/Si c) Raman spectrum of 1 μM MB /7 nm hBN film after cleaning in ethanol for 45 min at 10 positions. Apart from the hBN and Si peak no other peak can be seen. * For all the Raman measurements in this slide -532nm excitation, 100X objective, integration time -5s, Accumulation-2, input laser powers of 100 mW for (a) & (c) and 25 mW were used for data presented in for the data presented in (b).

9.

Table S2: Comparison of Raman enhancement of hBN films with other 2D materials

Sl. No	Substrate	Probe Molecule	Excitation wavelength (nm)	Laser Power (mW)	Enhancement Factor
**1.**	CVD Graphene on Ag nanoparticles over Si[1]	R6G	633	20	8.3×10^6
**2.**	Exfoliated hBN on Au nanoparticles[2]	R6G	514.5		2.5×10^5
**3.**	CVD hBN film **(Our work)**	MB	532	100	1.4×10^4
		MG	532	100	6.4×10^3
		R6G	532	25	1.5×10^4
**4.**	CVD hBN film + Au Nanoparticles **(Our work)**	MB	532	25	2.9×10^5
			633	10	1.6×10^6
**5.**	Graphene Flake[3]	CuPc	632.8	1	63
**6.**	hBN Flake [3]	CuPc	632.8	1	13
**7.**	MoS_2 Flake [3]	CuPc	632.8	1	16
**8.**	Monolayer MoS_2[4]	4-mercaptopyridine	488	0.6	3.8×10^5

References:

[1] X. Meng, H. Wang, N. Chen, P. Ding, H. Shi, X. Zhai, Y. Su, and Y. He, Anal. Chem. **90**, 5646 (2018).

[2] Q. Cai, S. Mateti, K. Watanabe, T. Taniguchi, S. Huang, Y. Chen, and L.H. Li, ACS Appl. Mater. Interfaces **8**, 15630 (2016).

[3] X. Ling, W. Fang, Y.H. Lee, P.T. Araujo, X. Zhang, J.F. Rodriguez-Nieva, Y. Lin, J. Zhang, J. Kong, and M.S. Dresselhaus, Nano Lett. **14**, 3033 (2014).

[4] C. Muehlethaler, C.R. Considine, V. Menon, W.C. Lin, Y.H. Lee, and J.R. Lombardi, ACS Photonics **3**, 1164 (2016).

10. Hybrid hBN and Au nanoparticles substrate

Au nanoparticles were deposited on 6 nm hBN film.

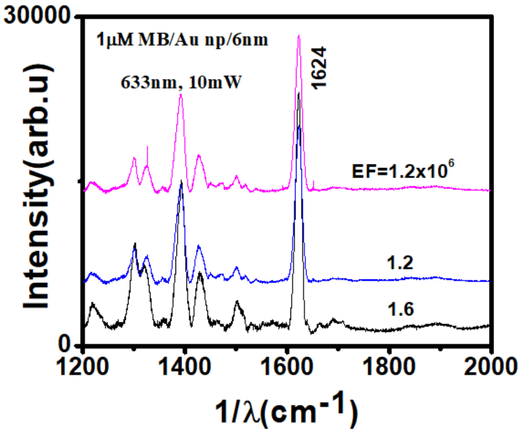

Figure S8: Raman enhancement of 1 micro molar MB with Au nano particles and hBN 633nm excitation with 10mW.

11. Regenerative and film durability study with the 6nm film- We performed enhancement studies with regenerating a 6nm film. The 1 μM MB/6 nm hBN was cleaned similarly like the 7nm film and after cleaning Raman spectrum was taken was different position (Fig.S7a), no MB peak can be seen. After re-drop casting with 1 μM MB we are able to achieve similar order of enhancement. The 6nm film was also employed to study the durability and reliability of the film for SERS if it is stored under ambient condition. At different span from their synthesis we did enhancement studies, which produced similar AEF.

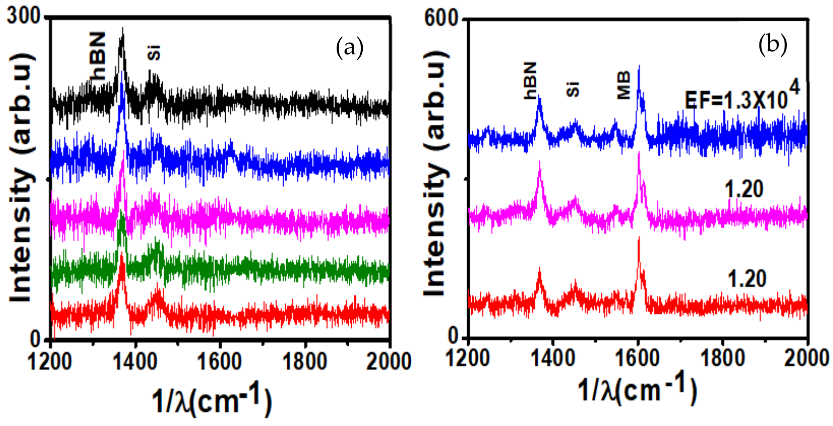

Figure S9: (a) Raman spectrum of the 1 μM MB/6 nm hBN after washing in ethanol for 45 min. (b) Re-drop casting 1 μM MB over the film in (a). Clearly regeneration has been achieved.

12. Simulation Details

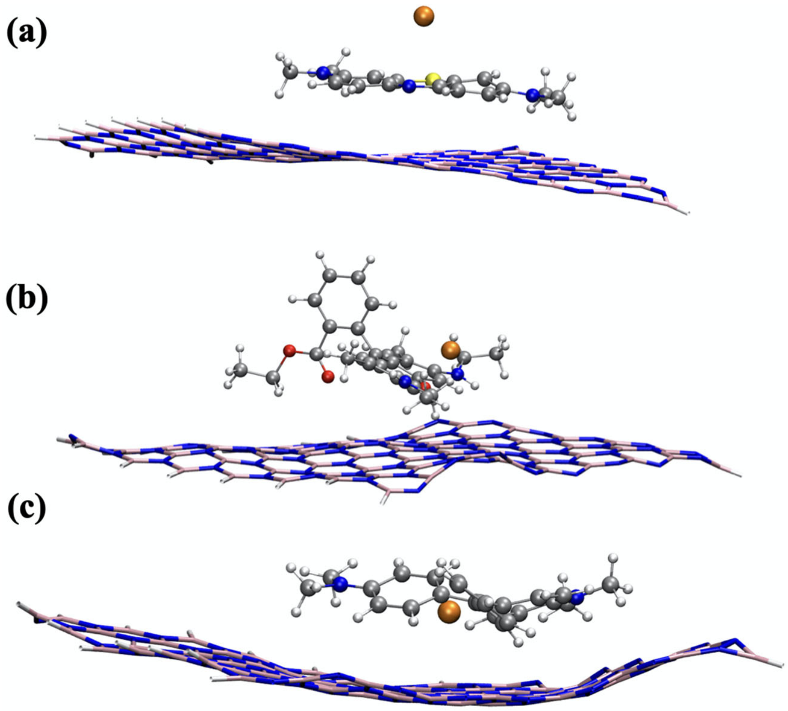

Figure S10: Full view of the optimized geometries of complexes of a single layer of hBN and dye (a) methylene blue (MB), (b) Rhodamine 6 G (R6G) and malachite green (MG).

13.

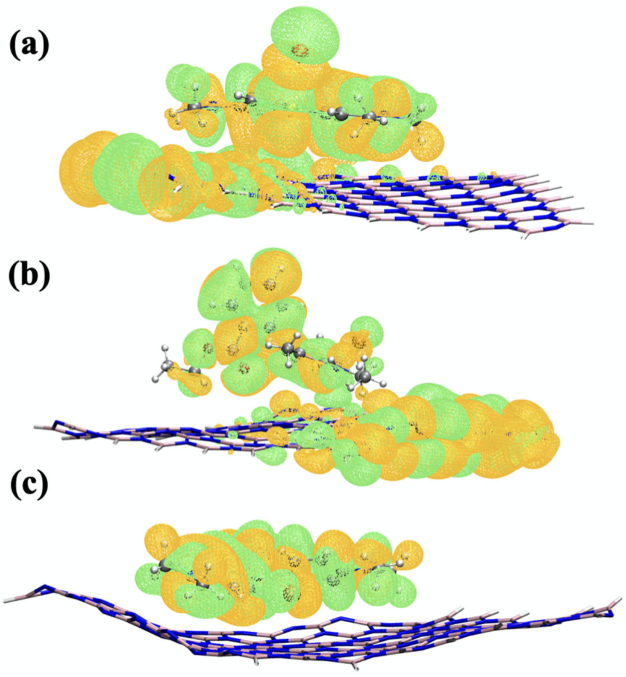

Figure S11: Full view of HOMO electron density surfaces for isodensity value of 0.007a.u. of the optimized geometries of complexes of a single layer of hBN and dye (a) methylene blue (MB), (b) Rhodamine 6G (R6G) and malachite green (MG).
